# Bismuth Complex Controlled Morphology Evolution and CuSCN-Induced Transport Improvement Enable Efficient BiI_3_ Solar Cells

**DOI:** 10.3390/nano12183121

**Published:** 2022-09-08

**Authors:** Zhangwei He, Runnan Yu, Wanrong Song, Yongshuai Gong, Hui Li, Zhan’ao Tan

**Affiliations:** 1 Beijing Advanced Innovation Center for Soft Matter Science and Engineering, Beijing University of Chemical Technology, Beijing 100029, China; 2 College of Materials Science and Engineering, Beijing University of Chemical Technology, Beijing 100029, China

**Keywords:** bismuth triiodide, coordination engineering strategy, morphology evolution, charge transport, inorganic solar cells

## Abstract

Bismuth triiodide (BiI_3_) is a particularly promising absorber material for inorganic thin-film solar cells due to its merits of nontoxicity and low cost. However, one key factor that limits the efficiency of BiI_3_ solar cells is the film morphology, which is strongly correlated with the trap states of the BiI_3_ film. Herein, we report a coordination engineering strategy by using Lewis base dimethyl sulfoxide (DMSO) to induce the formation of a stable BiI_3_(DMSO)_2_ complex for controlling the morphology of BiI_3_ films. Density functional theory calculations further provide a theoretical framework for understanding the interaction of the BiI_3_(DMSO)_2_ complex with BiI_3_. The obtained BiI_3_(DMSO)_2_ complex could assist the fabrication of highly uniform and pinhole-free films with preferred crystallographic orientation. This high-quality film enables reduced trap densities, a suppressed charge recombination, and improved carrier mobility. In addition, the use of copper(I) thiocyanate (CuSCN) as a hole transport layer improves the charge transport, enabling the realization of solar cells with a record power conversion efficiency of 1.80% and a champion fill factor of 51.5%. Our work deepens the insights into controlling the morphology of BiI_3_ thin films through the coordination engineering strategy and paves the way toward further improving the photovoltaic performances of BiI_3_ solar cells.

## 1. Introduction

Inorganic metal halide material seems to be one of the most promising light absorbers for low-cost, eco-friendly, next-generation solar cells owing to its superior stability and potentially high efficiency [1,2,3,4]. Bismuth triiodide (BiI_3_), as a novel light absorber inorganic metal halide material, has attracted great interest due to its low-toxicity, earth-abundance and good optoelectrical properties. The absorption coefficient of BiI_3_ (>10^5^ cm^−1^) is competitive with that of Si and metal halide perovskite in the visible region of the solar spectrum [5,6]. In addition, the electron diffusion length and electronic mobility of the BiI_3_ can reach 4.9 μm and 600 cm^2^ V^−1^ s^−1^, respectively, which are comparable with the properties of typical thin-film materials, such as CdTe [7,8,9,10]. These features indicate that BiI_3_ is a promising absorber material, and the first BiI_3_ solar cell was demonstrated in 2015 with a power conversion efficiency (PCE) of 0.3% [11]. Obviously, the efficiency is pretty low and further improvement is greatly needed to solve the challenges that hamper the development of BiI_3_ solar cells.

One of the most important challenges is the acquisition of high quality BiI_3_ thin films with appropriate morphology, which is closely related to the optoelectronic properties of BiI_3_ films. To obtain dense and uniform absorber films for fabricating high-performance BiI_3_ solar cells, various methodologies have been developed to optimize the morphology of BiI_3_ thin films. Post-treatment including recycled vapor annealing [12], thermal annealing [13], and solvent vapor annealing [14], and precursor engineering including additive engineering [15], solvent engineering [16], and coordination engineering [17], are feasible methods to promote crystal growth and improve the crystallinity of BiI_3_ thin films. Coordination engineering has been proven to be an effective strategy in lead-based perovskite solar cells to control the morphology of absorber layers by changing the coordination solvent [18,19,20,21]. Since both Pb and Bi halides can form adducts with most Lewis base solvents, one can utilize a coordination engineering strategy to manipulate the crystallization dynamics and the resulting morphology of BiI_3_ absorber layers by varying the concentration of Lewis base solvents in precursor solutions to tune the interaction strength between BiI_3_ and Lewis base solvents. For example, the preferred orientation, aggregate size, and surface coverage of the BiI_3_ thin film was finely tuned by controlling the mixing ratios of solvent additive with a higher Gutmann donor number [17]. However, a scientific investigation of the status of coordination complexes during the film fabrication process has not been thoroughly conducted, and fundamental mechanisms in the complexes’ formation to manipulate grain nucleation and growth have not been well addressed yet.

In addition, another important challenge is how to construct efficient devices based on the high quality BiI_3_ thin films. NbSex interlayers were inserted between the poly(3,4-ethylenedioxythiophene)–poly(styrenesulfonate)(PEDOT:PSS) buffer layer and the BiI_3_ active layer to decrease the interfacial recombination and enhance exciton separation, and the PCE of the device increased from 0.33 to 0.64% [22]. An in situ-generated bismuth sulfide iodide (BiSI) interlayer at the interface of the BiI_3_ absorber layer and the SnO_2_ electron transport layer by the reaction of In_2_S_3_ and BiI_3_ at 200 °C were introduced in ITO/SnO_2_/BiSI/BiI_3_/Spiro-OMeTAD/Au structured solar cells, and a PCE of 1.21% was achieved due to the improved charge separation [23]. Highly crystalline BiI_3_ films with a rhombohedral phase and a high-degree of stacking order were obtained through gas-phase iodination of Bi_2_S_3_ from thermolysis of Bi(NO_3_)_3_ and thiourea precursor. By introducing the polymer hole transport layer (HTL), poly(9,9-di-n-octylfluorenyl-2,7-diyl) (F8), an open-circuit voltage (*V*_OC_) of 0.6 V and a PCE of 1.2% were achieved for FTO/TiO_2_/BiI_3_/F8/Au-based solar cells [24]. We previously introduced a light-absorbing conjugated polymer for building a BiI_3_/polymer heterojunction to expand the light harvesting, and the highest short-circuit density (*J*_SC_) of 7.8 mA cm^−2^ was achieved for BiI_3_-based solar cells with light response to 800 nm [15]. Thus far, the record PCE of 1.5% was achieved for BiI_3_ solar cells by constructing a binary quasi-bulk heterojunction between a BiI_3_ electron donor and PC_61_BM electron acceptor, which effectively promoted the exciton separation [25]. 

In this work, we report a coordination engineering strategy by introducing a Lewis base dimethyl sulfoxide (DMSO) into the BiI_3_ precursor solution to form a stable complex BiI_3_(DMSO)_2_, which favors control of the crystallization processes of BiI_3_ and manipulates the morphology of the BiI_3_ layer. The crystalline structure of BiI_3_(DMSO)_2_ was confirmed by single-crystal X-ray diffraction, and further density functional theory (DFT) calculations proved that the formed BiI_3_(DMSO)_2_ tends to fill the iodine vacancies, resulting in a strong interaction between the BiI_3_(DMSO)_2_ complex and the BiI_3_ active layer. This high-quality film enables reduced trap densities, suppressed charge recombination, and improved carrier mobility. We further employed copper(I) thiocyanate (CuSCN) as a HTL to fabricate BiI_3_ solar cells. CuSCN exhibits a chemical compatible and deeper-lying valence band maximum (VBM) with the valence band of BiI_3_, leading to improved carrier transfer and good Ohmic contact. Benefiting from the high-quality BiI_3_ film and excellent hole transport of CuSCN, a BiI_3_ solar cell with a structure of ITO/CuSCN/BiI_3_/PC_71_BM/Ca/Al is demonstrated, and a record PCE of 1.80% with a champion fill factor (FF) of 51.5% were achieved.

## 2. Materials and Methods

### 2.1. Materials

Indium tin oxide (ITO) glass substrates were purchased from South China Science & Technology Company Limited (Shenzhen, China). Anhydrous bismuth triiodide (BiI_3_, >98.0%, anhydrous) was purchased from Tci (Shanghai, China). Copper(I) thiocyanate (CuSCN, 99%) was purchased from Aladdin (Shanghai, China). [6,6]-phenyl-C71-butyric acid methyl ester (PC_71_BM) was purchased from Organtec Ltd (Beijing, China). Diethyl sulfide (DES, 97%+) was purchased from Adamas (Shanghai, China). Tetrahydrofuran (THF, 99.5%, extra dry), dimethyl sulfoxide (DMSO, 99.7+%, extra dry), trichloromethane (CF), and chlorobenzene (CB, 99.8%, extra dry) were purchased from Acros (Geel, Belgium). All of the materials were used as received without further purification.

### 2.2. Preparation of Precursor Solution

For the BiI_3_ solution, 120 mg/mL BiI_3_ solution was prepared by dissolving BiI_3_ in THF. The Lewis base solvent DMSO was added to the THF in the following volume ratios: 0%, 0.3%, 0.5%, and 0.7%. For the CuSCN solution, 25 mg/mL CuSCN solution was prepared by dissolving CuSCN in DES. The BiI_3_ solution and the CuSCN solution were stirred at 1000 rpm overnight at room temperature. Afterwards, the BiI_3_ solution and the CuSCN solution were filtered with a 0.22 µm PTFE filter and 0.2 µm PVDF filter, respectively. For the PC_71_BM solution, 15 mg/mL PC_71_BM solution was prepared by dissolving PC_71_BM in CB. The PC_71_BM solution was stirred at 800 rpm overnight at 40 °C.

### 2.3. Device Fabrication

ITO glass substrates were cleaned with detergent, water, ultrapure water, and ethanol for 15 min in an ultrasonicator, respectively. Next, the substrates were treated with UV-ozone for 15 min before use. Then, the CuSCN thin films were deposited as HTL by spin-coating at 3500 rpm for 60 s and were annealed at 100 °C for 10 min. The BiI_3_ solution with or w/o DMSO was coated by spin-coating on the CuSCN substrates at 3000 rpm for 30 s; 150 µL of CF was dropped on the spinning substrate 20 s prior to the end of the program. Afterward, the substrates were transferred onto a hotplate and heated at 100 °C for 10 min. After the substrates cooled down to room temperature, a PC_71_BM solution was spin-coated at 1300 rpm for 60 s. Then the substrates were heated at 100 °C for 10 min. Finally, 20 nm Ca and 100 nm Al was deposited on the ETM surface by thermal evaporation.

### 2.4. Characterization

Ruby-red crystals of BiI_3_(DMSO)_2_ were prepared by means of the previously reported anti-solvent vapor-assisted crystallization approach [26], using 1.0 mL of a saturated DMSO solution of BiI_3_ with CHCl_3_ as the anti-solvent. The crystal structure of BiI_3_(DMSO)_2_ was characterized by single-crystal X-ray diffraction (Rigaku XtalAB PRO MM007DW, Tokyo, Japan). Direct methods and SHELXTL program were employed to solve and refine the crystal structure. The chemical composition of BiI_3_ films was analyzed applying X-ray photoelectron spectroscopy (XPS, Kratos AXIS ULTRA DLD, Kyoto, Japan). A Fourier transform infrared spectroscopy (FTIR, Nicolet 8700, Thermo Electron Corporation, Waltham, MA, USA) was employed to obtain the FTIR spectral data for DMSO (liquid phase) and bismuth complexes. The crystal structures of the formed BiI_3_ films were characterized by performing X-ray powder diffractometer (XRD-6000, SHIMADZU, Kyoto, Japan). Thermogravimetric analysis (TGA) was performed using a TA Instruments TGAQ500 (New Castle, PA, USA) with a ramp of 10 °C min^–1^ under N_2_ from 30 to 800 °C. A UV–Vis–NIR 3600 spectrometer (SHIMADZU, Kyoto, Japan) was performed to obtain UV–Vis spectroscopy. The morphology of BiI_3_ films was characterized by conducting scanning electron microscopy (SEM, HITACHI S-470, Tokyo, Japan) and atomic force microscope (AFM, Bruker DMFASTSCAN2-SYS, Karlsruhe, Germany). The electronic properties of bismuth complex and CuSCN HTL films were characterized by ultraviolet photoemission spectroscopy (UPS, Kratos AXIS ULTRA DLD, He−Iα = 21.22 Ev, Kyoto, Japan). The *J*–*V* characteristics were measured under the illumination with a solar simulator (SS-F5-3A, EnliTech, Kaohsiung City, Taiwan) at an intensity 100 mW/cm^2^ in N_2_ atmosphere. The EQE measurements were performed using QE-R systems (EnliTech, Kaohsiung City, Taiwan) in ambient atmosphere. 

### 2.5. Computational Methods

The DFT calculations were performed with PAW pseudopotential method as implemented in the Vienna Ab Initio Simulation Package (VASP) [27,28]. The Perdew–Burke–Ernzerhof (PBE) functional within the generalized gradient approximation (GGA) exchange correlation was utilized [29]. The vdW correction of Grimmer’s DFT+D3 was included in all calculations [30,31] because the vdW correction plays an important role in describing the weak interactions within perovskite material. The plane wave cutoff was set to 400 eV for the adsorption of BiI_3_ (DMSO)_2_ on the BiI_3_ surfaces. The 1 × 1 × 1 k-point mesh was used to optimize the structure in the 3 × 3 supercell. In all calculations, the value of vacuum space is about 15 Å along the z direction to eliminate the image interaction, and the structures were relaxed until the maximum atomic force was less than 0.01 eV·Å^−1^. The energy difference of these structures was within 10^−5^ eV. The adsorption energies of BiI_3_ (DMSO)_2_ on the surface of BiI_3_ were calculated as follows

*E*_ads_ = *E*(BiI_3_ + complex) − *E*(complex) − *E*(BiI_3_)

where *E*(BiI_3_ + complex) represents the total energy of the BiI_3_(DMSO)_2_ complex on the BiI_3_ substrates, *E*(BiI_3_) is the energy of the BiI_3_ substrates, and *E*(complex) is the energy of the BiI_3_(DMSO)_2_ complex.

## 3. Results and Discussion

**Figure 1:**
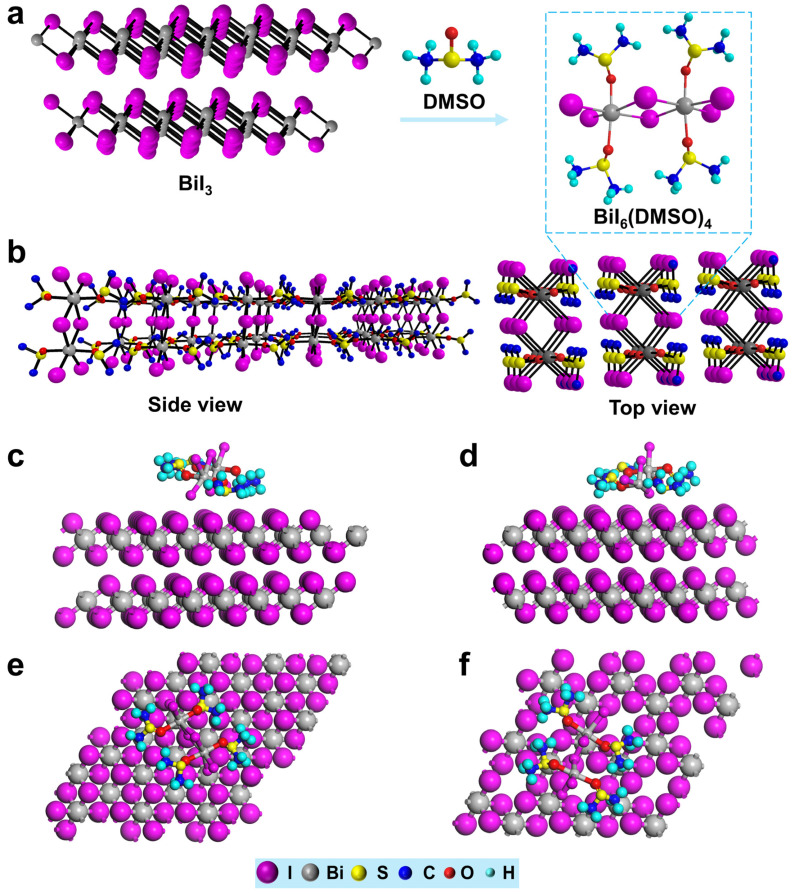
(**a**) Schematic diagram on the formation process and (**b**) crystal structure of BiI_3_(DMSO)_2_. The boxed fragment Bi_2_I_6_(DMSO)_4_ has a space group P1 with dimensional parameters a = 8.3303(6) Å, b = 8.8638(6) Å, c = 12.4967(8) Å, α = 92.275(5)°, β = 101.467(6)°, γ = 117.263(7)°. DFT calculations. (**c**) The front view and (**e**) the top view of BiI_3_(DMSO)_2_ complex absorbs on the BiI_3_ surfaces. (**d**) The front view and (**f**) the top view of BiI_3_(DMSO)_2_ complex adsorbs on the defective BiI_3_ surface containing iodide vacancies.

Figure 1a shows the crystals of BiI_3_(DMSO)_2_, which can be obtained by the coordination engineering strategy of introducing Lewis base DMSO into the BiI_3_ precursor solution followed by an anti-solvent vapor-assisted crystallization approach [26]. Figure 1b gives the crystal structure of BiI_3_(DMSO)_2_, which is further confirmed by single-crystal X-ray diffraction and the structural details are listed in Table S1. The incorporation of a DMSO ligand changes the coordination state of BiI_3_. The boxed fragment can be described as a symmetric complex (Bi_2_I_6_(DMSO)_4_) with an edge-shared and bi-octahedral crystal structure. Six iodide ions occupy the corners of the bi-octahedral, while the Bi ions at the body center and the O-coordinated DMSO molecules complete the coordination structure. 

To gain insight into the interaction between the BiI_3_(DMSO)_2_ complex and BiI_3_ surfaces, first-principles DFT calculations were carried out (the procedure is outlined in the Experimental Section in the Supporting Information). The optimized structures of BiI_3_(DMSO)_2_ absorbing on the BiI_3_ surfaces are shown in Figure 1c–f. In order to visualize the adsorption structure, only the upper adsorption structures are presented. The adsorption energy of BiI_3_(DMSO)_2_ on the BiI_3_ is −1.08 eV. Structural defects are often the most important factor affecting the performance of solar cell materials, so a full understanding of the defect is of great importance. The iodide vacancy defect was introduced to BiI_3_ surfaces due to its low formation energy. Comparing the perfect BiI_3_ surfaces, the adsorption energy of the BiI_3_(DMSO)_2_ complex on defective BiI_3_ surfaces changes to −1.83 eV, which suggests a stronger adsorption on the defective BiI_3_ surfaces than the perfect case. When iodide vacancies within BiI_3_ surfaces are created, the iodine element in the BiI_3_(DMSO)_2_ complex tends to fill the iodine vacancies, resulting in a significant interaction between the BiI_3_(DMSO)_2_ complex and the defective BiI_3_ surfaces. Overall, our theoretical calculation results confirm that the adsorption of the BiI_3_(DMSO)_2_ complex successfully passivates the BiI_3_ film to modulate its surfaces’ properties.

**Figure 2:**
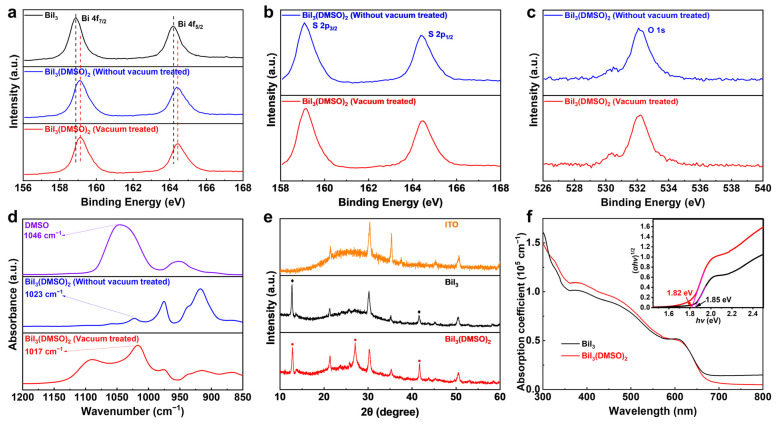
High-resolution XPS spectra for (**a**) Bi 4f of BiI_3_ film, DMSO-coordinated BiI_3_ film w/o and with vacuum treatment. (**b**) S 2p and (**c**) O 1s of DMSO-coordinated BiI_3_ film w/o and with vacuum treatment. (**d**) FTIR of pure DMSO, DMSO-coordinated BiI_3_ film w/o and with vacuum treatment. (**e**) XRD patterns of ITO substrate, BiI_3_ films, and DMSO-coordinated BiI_3_ films. (**f**) UV−Vis absorption spectra of BiI_3_ films w/o and with DMSO. Inset shows the Tauc analysis of the absorption spectra.

To deeply understand the bonding interactions between BiI_3_ and DMSO, the X-ray photoelectron spectroscopy (XPS) characterizations were performed. The survey scans of BiI_3_ and DMSO-coordinated BiI_3_ are shown in Figure S1. The core level scans of Bi 4f for each sample are compared (Figure 2a). For the BiI_3_ film, two main peaks located at 164.2 and 158.9 eV are observed, which are assigned to Bi 4f_5/2_ and Bi 4f_7/2_, respectively. However, in the DMSO-coordinated sample, the peaks of Bi 4f shift to a higher binding energy compared to that of BiI_3_ film, attributed to the formation of coordination bonds between Bi from the BiI_3_ and O from DMSO. More importantly, it can be seen that no significant shift in the Bi 4f core level is observed after vacuum treatment for the DMSO-coordinated BiI_3_ film under a pressure of 5 × 10^−5^ Pa for 1 h. In addition, the existence of S and O is proved by the evident signals of S 2p at 159.1 and 164.4 eV and O 1s at 532.2 eV (Figure 2b,c). The stability of the BiI_3_(DMSO)_2_ complex was further confirmed by TGA measurement. The TGA curves in Figure S2 revealed that the BiI_3_(DMSO)_2_ complex was thermally stable up to 190 °C. These results confirm that BiI_3_(DMSO)_2_ acts as a stable complex rather than an intermediate adduct phase. 

The coordination interaction was further confirmed by Fourier transform infrared spectroscopy (FTIR). As shown in Figure 2d, the stretching vibration of S=O (ν_(S=O)_) appears at 1046 cm^−1^ for the pure DMSO, which is shifted to 1023 cm^−1^ upon formation of the BiI_3_(DMSO)_2_ complex [20,32,33]. According to the diatomic harmonic model, the square root of the force constant is proportional to the frequency of vibration [34]. Based on this model, the decreased S=O stretching vibration frequency denotes that the force constant is reduced, which is ascribed to the decreased strength of the S=O bond as a consequence of BiI_3_(DMSO)_2_ complex formation. Therefore, the S=O stretching vibration frequency of BiI_3_(DMSO)_2_ is detected in a lower wavenumber than that of DMSO. Additionally, the stretching vibration frequency of S=O does not shift after vacuum treatment, indicating the strong interaction between BiI_3_ and DMSO, which is consistent with the observation from XPS. 

The effects of the BiI_3_(DMSO)_2_ complex on the crystallographic structure and optical properties of BiI_3_ films were further investigated. The X-ray diffraction (XRD) patterns of BiI_3_ films processed in different conditions are shown in Figure 2e. The BiI_3_ film processed without DMSO exhibits two diffraction peaks at 2-Theta of 12.8° and 41.6°, which are in good accordance with previous work [14]. Notably, in the DMSO-coordinated BiI_3_ film, a signature peak at 26.9° appears, indicating the preferred orientation of the BiI_3_(DMSO)_2_ complex on the (113) lattice plane. It can be concluded that the DMSO-coordinated BiI_3_ thin film is composed of the BiI_3_(DMSO)_2_ complex and BiI_3_ from the results of XPS and XRD spectra. This is completely consistent with the DFT calculation. UV−Vis absorption spectra (Figure 2f) were measured to evaluate the effect of the formation of the BiI_3_(DMSO)_2_ complex on the optical properties of BiI_3_ films. The absorption of BiI_3_ film in the wavelength region from 300 to 650 nm is slightly enhanced with the addition of DMSO, and Tauc analysis of the absorption spectra reveals a slightly decreased band gap from 1.85 to 1.82 eV for the DMSO-coordinated BiI_3_ film (Figure 2f, inset). These results suggest that the formation of BiI_3_(DMSO)_2_ can significantly affect the crystallization kinetics of BiI_3_ and change the optoelectronic properties of the thin film.

**Figure 3:**
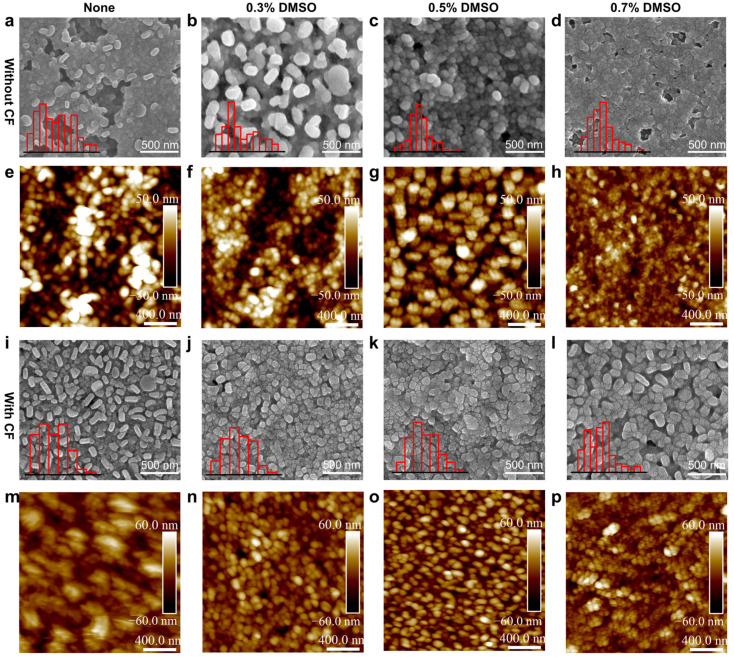
SEM (**a**–**d**,**i**–**l**) and AFM (**e**–**h**,**m**–**p**) images of BiI_3_ films with different volume percentage of DMSO without and with CF anti-solvent treatment.

In order to further understand the influences of DMSO concentrations and CF anti-solvent treatment on the crystallization process and relevant thin film morphology, top-view scanning electron microscopy (SEM) and atomic force microscopy (AFM) measurements on the corresponding BiI_3_ thin films were conducted. As shown in Figure 3a, the as-cast BiI_3_ film without DMSO exhibits incomplete surface coverage and is composed of rod-like BiI_3_ crystals, which can be ascribed to a relatively fast crystal growth rate during the spin-coating process. After adding 0.3% DMSO, the uniformity of BiI_3_ film is significantly improved, while there exists some small void spaces (Figure 3b). As depicted in Figure 3c, the 0.5% DMSO-coordinated film shows a compact and uniform morphology. These could be attributed to the fact that the BiI_3_(DMSO)_2_ complex controls nucleation, retards the crystal growth, and then assists the formation of a highly uniform morphology. When the content of DMSO further increases to 0.7%, some pinholes appear, while most regions of the film are still quite dense (Figure 3d). Interestingly, when we used CF as the anti-solvent, the quality of the obtained film was significantly improved compared with that of the film without dropping anti-solvent. It can be seen from Figure 3i–l that a dense and uniform BiI_3_ film morphology can be obtained after CF anti-solvent treatment. The control BiI_3_ film shows a smooth morphology with an apparent grain boundary, while the DMSO-coordinated one displays an increased crystal domain size and more uniform size distribution than the control sample. The uniformity and compactness further increase at higher DMSO loading. The influences of DMSO concentrations and CF anti-solvent treatment on the BiI_3_ surface roughness were monitored by AFM (Figure 3e–h,m–p). The crystal domain size increases after the introduction of DMSO coordinative solvent and CF anti-solvent. This is consistent with the results obtained from SEM. The measured root-mean-square roughness values of the control film and DMSO-coordinated BiI_3_ film with CF anti-solvent are 15.0 nm and 18.1 nm, respectively. The larger and more uniform crystal grain size of the thin film suggests reduced defects as well as nonradiative recombination centers. These results demonstrated that the utilization of CF as an anti-solvent and DMSO as a coordinative solvent successfully assist the formation of a uniform and compact absorber layer.

**Figure 4:**
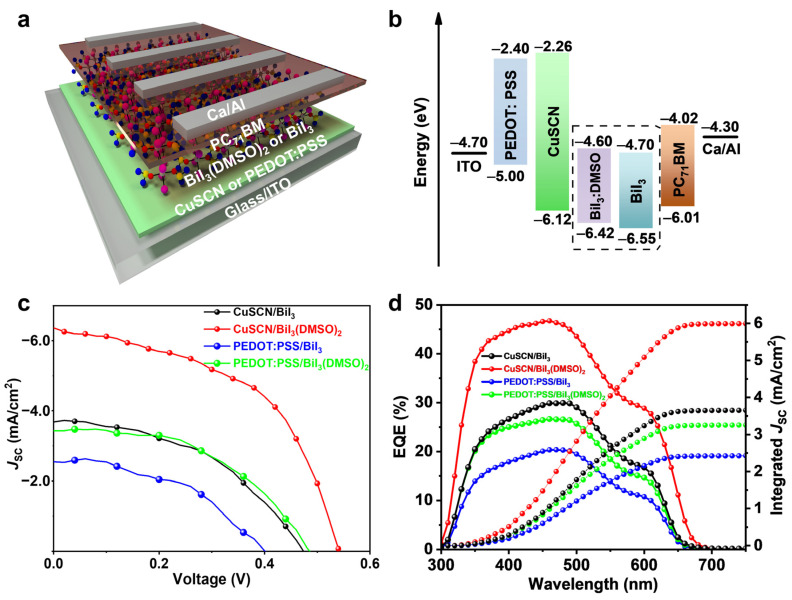
(**a**) p–i–n-structured planar heterojunction solar cells with CuSCN or PEDOT:PSS as HTL and BiI_3_ or DMSO-coordinated BiI_3_ as photoactive layer. (**b**) The energy level diagram of the p–i–n-structured devices. (**c**) *J*–*V* curves and (**d**) EQE spectrum of the corresponding solar cells.

Based on the obtained high quality BiI_3_ thin films, p–i–n structured planar heterojunction solar cells were designed and fabricated (Figure 4a). For this structure, PEDOT:PSS is the most commonly used HTL [13,22,25]. However, the PEDOT:PSS exhibits a shallow VBM, which is a mismatch with the deep-lying valence band of BiI_3_. Considering the energetic alignment theory, the HTL should have a relatively deep VBM to match with that of the BiI_3_ absorber layer. Fortunately, inorganic CuSCN with a deeper lying VBM can perfectly meet this demand as the HTL, and the good alignment with the BiI_3_ film can greatly enhance the hole extraction. The energy levels of CuSCN and BiI_3_ films were determined from ultraviolet photoelectron spectroscopy (UPS) and optical measurements of the band gap (Figures S3 and S4). Figure 4b gives the energy level diagram of the device with the structure of ITO/CuSCN/BiI_3_/PC_71_BM/Ca/Al (Figure 4a). The valence band edge for CuSCN is 6.12 eV, which is close to that of the BiI_3_ film (6.55 eV) and DMSO-coordinated BiI_3_ film (6.42 eV). However, for PEDOT:PSS with a HOMO level of 5.0 eV, there is a large barrier of over 1.42 eV to the photoactive layer of BiI_3_ and DMSO-coordinated BiI_3_ films. Obviously, more favorable energetics alignment with a CuSCN HTL can be achieved in comparison to PEDOT:PSS-based devices. 

For comparison, BiI_3_-based solar cells with CuSCN or PEDOT:PSS as the HTL and BiI_3_ or DMSO-coordinated BiI_3_ as the photoactive layer were fabricated. Figure 4c gives the current density–voltage (*J*–*V*) curves of the corresponding devices under AM 1.5 G (100 mW/cm^2^) illumination, and the photovoltaic parameters of the champion devices are summarized in Table 1. The PEDOT:PSS-based device with a BiI_3_ photoactive layer shows a *V*_OC_ of 0.40 V, an FF of 46.8%, a *J*_SC_ of 2.54 mA/cm^2^ and a PCE of 0.48%. By using DMSO-coordinated BiI_3_ as the photoactive layer, all the photovoltaic parameters are increased, achieving a PCE of 0.81%, with an FF of 48.6%, a *V*_OC_ of 0.49 V, and a *J*_SC_ of 3.43 mA/cm^2^. Moreover, introducing CuSCN as the HTL and BiI_3_ as the photoactive layer, the device shows a PCE of 0.80%, with a *V*_OC_ of 0.47 V, an FF of 45.9%, and a *J*_SC_ of 3.68 mA/cm^2^. Further employing DMSO-coordinated BiI_3_ as the photoactive layer, all photovoltaic parameters can be simultaneously improved and the best PCE of 1.80% can be achieved, with an FF of 51.5%, a *V*_OC_ of 0.55 V, and a *J*_SC_ of 6.38 mA/cm^2^. Compared with previous reports, the achieved PCE of 1.80% and FF of 51.5% are the highest values reported for BiI_3_ solar cells (Figure S5 and Table S2), which is related to the device structure and morphology of BiI_3_ thin films [11,12,13,14,15,16,17,22,23,24,25,35]. Haque et al. used SnO_2_ to fabricate n-i-p BiI_3_ solar cells, which resulted in a *J*_SC_ of up to 12.6 mA/cm^2^ and PCE of 1.21%; however, the FF remained limited to 29.0% [23]. Conducting polymers such as polytriarylamine (PTAA) has been employed as an HTL [13]. The HTL can improve the *V*_OC_ but at the cost of the *J*_SC_. CuSCN in our devices can improve the charge transfer and the BiI_3_(DMSO)_2_ complex can control the quality of BiI_3_ films. Notably, our champion BiI_3_ solar cell reached a relatively higher PCE in Bi-based solar cells (Table S3) [36,37,38,39,40,41]. Figure 4d displays the external quantum efficiency (EQE) spectra of the corresponding devices, and the integrated *J*_SC_ is in good agreement with the *J–V* curve-derived *J*_SC_, showing the strong reliability of the tested results. We notice that the *V*_OC_ values of CuSCN-based devices are higher than those of their PEDOT:PSS-based counterparts due to the better VBM alignment of the CuSCN HTL with the BiI_3_ photoactive layer (Figure 4b). To verify the reproducibility, a batch of 30 devices with a CuSCN HTL and BiI_3_ or DMSO-coordinated BiI_3_ photoactive layers was fabricated. The statistical photovoltaic parameters of *V*_OC_, *J*_SC_, FF, and PCE are given in Figure S6, and their performance features are summarized in Table S4. DMSO-coordinated BiI_3_-based devices show a narrow distribution with a high PCE of 1.75 (±0.02)%.

**Figure 5:**
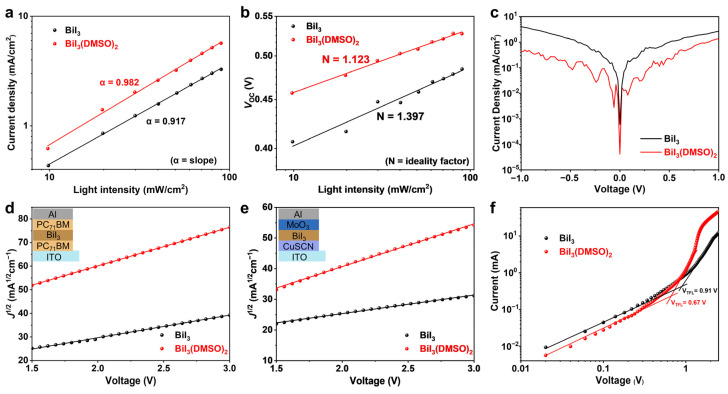
Light intensity-dependent (**a**) *J*_SC_, (**b**) *V*_OC_, and (**c**) dark *J*–*V* curves of devices with BiI_3_ and DMSO-coordinated BiI_3_ film. (**d**) Electron mobility, (**e**) hole mobility, and (**f**) trap density calculated from *J–V* curves of single-carrier devices with BiI_3_ and DMSO-coordinated BiI_3_ film.

To further understand the enhanced *J*_SC_ and FF for devices based on the DMSO-coordinated BiI_3_ photoactive layer, the light intensity-dependent *J*_SC_ and *V*_OC_ for both devices with BiI_3_ and DMSO-coordinated BiI_3_ were tested to investigate the charge recombination processes [42]. The *J*_SC_ versus light intensity (*P*_*light*_) follows the relationship of $\ln\left(J_{SC}\right)\propto \alpha \ln(P_{light})$, and the slop α reflects the charge recombination in the devices. As shown in Figure 5a, the device based on the DMSO-coordinated BiI_3_ film shows a slope of 0.982, while the control device with BiI_3_ film shows a smaller slope of 0.917. The larger slope for the DMSO-coordinated device indicates that the carrier recombination is suppressed, and the charge accumulation within the devices is prevented [43]. Figure 5b shows the relationship between *V*_OC_ and *P*_*light*_, which follows the relationship of $V_{OC}\propto N\left(K_{B}T/q\right)\ln\left(P_{light}\right)$ [44], where *q* is the elementary charge, *K*_B_ is the Boltzmann constant, and *T* is the temperature, and the charge recombination process is reflected by the ideality factor *N*. In general, the value of *N* approaches unity for ideal photovoltaic devices. However, when the value of *N* approaches 2, the Shockley−Read−Hall (SRH) recombination assisted by trap density dominates [45]. It can be seen that the *N* for the control device with the BiI_3_ layer is 1.40, while the device with the DMSO-coordinated BiI_3_ layer shows a smaller *N* of 1.12, indicating that the trap-assisted SRH recombination is effectively suppressed, and the inherent trap density is reduced. 

The dark current characteristics of BiI_3_ solar cells were measured to analyze the loss of the charge carrier via the leakage pathways and charge carrier recombination [46], and the related *J*−*V* curves are depicted in Figure 5c. The dark current significantly decreases in the DMSO-coordinated BiI_3_ device, denoting more charge carriers sweep through the device instead of shunting [47]. The decreased leakage current and suppressed carrier recombination result in the improvement in *J_SC_* and FF. Furthermore, the electron-only and hole-only devices (inset of Figure 5d,e) based on BiI_3_ and DMSO-coordinated BiI_3_ films were fabricated, and the hole and electron mobilities were calculated through the space-charge-limited current (SCLC) method [48]. The BiI_3_-based device gives a hole mobility of 3.98 × 10^−5^ cm^2^/Vs and electron mobility of 9.97 × 10^−5^ cm^2^/Vs. Contrastively, the DMSO-coordinated BiI_3_ device demonstrates much higher and more balanced hole and electron mobilities of 2.11 × 10^−4^ cm^2^/Vs and 2.90 × 10^−4^ cm^2^/Vs, respectively, which contribute to the enhancement of FF and *J*_SC_. 

To confirm the effect of the bismuth complex on the trap states within the BiI_3_ film, we estimated the trap density of BiI_3_ film by measuring dark current–voltage characteristics of the hole-only device, where the bias voltage determined as the trap-filled limited voltage (*V*_TFL_) is closely related with the trap density [49,50]. As given in Figure 5f, the measured *V*_TFL_ for the BiI_3_-based device is ~0.91 V, while the value decreased to 0.67 V for the DMSO-coordinated BiI_3_ device. The calculated trap density for the BiI_3_ film is 1.68 × 10^17^ cm^–3^ and for the DMSO-coordinated BiI_3_ film is 1.23 × 10^17^ cm^–3^. The reduced trap density in the DMSO-coordinated BiI_3_ film can effectively suppress the nonradiative recombination, resulting in an improved FF [51,52]. 

## 4. Conclusions

In conclusion, we successfully demonstrate efficient BiI_3_ solar cells by controlling BiI_3_ film morphology via a stable BiI_3_(DMSO)_2_ complex and introducing CuSCN as the HTL for transport improvement. DFT calculation reveals that BiI_3_(DMSO)_2_ can fill the iodide vacancies in the BiI_3_ film to modulate its surface properties. The obtained BiI_3_(DMSO)_2_ could trigger homogeneous nucleation and enable a slow crystal growth rate, inducing the formation of highly uniform and pinhole-free BiI_3_ films with preferred crystallographic orientation and enhanced optical absorption. This high-quality film enables reduced trap densities, suppressed charge recombination, and improved carrier mobility. Furthermore, a deeper lying VBM of the CuSCN HTL ensures Ohmic contact and good charge transport with the BiI_3_ layer. Overall, a record PCE of 1.80% with a champion FF of 51.5% were achieved for BiI_3_ solar cells.

## Figures and Tables

**Table 1:** Photovoltaic parameters of solar cells with CuSCN or PEDOT:PSS as HTL and BiI_3_ or DMSO-coordinated BiI_3_ as photoactive layer.

Hole Transport Layer	Active Layer	*J*_SC_ (mA/cm^2^)	*V*_OC_ (V)	FF (%)	PCE (%)
PEDOT:PSS	BiI_3_	2.54	0.40	46.8	0.48
PEDOT:PSS	BiI_3_:DMSO	3.43	0.49	48.6	0.81
CuSCN	BiI_3_	3.68	0.47	45.9	0.80
CuSCN	BiI_3_:DMSO	6.38	0.55	51.5	1.80

## Data Availability

The data presented in this study are available on request from the corresponding author.

## Supplementary Materials

The following supporting information can be downloaded at: https://www.mdpi.com/article/10.3390/nano12183121/s1, Figure S1: XPS spectra for BiI_3_ film, and the DMSO-coordinated BiI_3_ film without and with vacuum treatment; Figure S2: Thermogravimetric analysis of BiI_3_(DMSO)_2_ at (scan rate: 10 °C min^−1^); Figure S3: UPS spectra of (a,c,e) the high-binding energy secondary electron cutoff regions and (b,d,f) the valence band edge regions of BiI_3_ film with and w/o DMSO-treated and CuSCN film, respectively; Figure S4: UV−Vis absorption spectra of CuSCN; Figure S5: Comparison of PCE and FF values of this work with previous reported BiI_3_ photovoltaic devices; Figure S6: Statistical photovoltaic parameters obtained from 30 photovoltaic devices with CuSCN HTL and BiI_3_ or DMSO-coordinated BiI_3_ photoactive layers; Table S1: Crystal data and structure refinement for BiI_3_(DMSO)_2_ at 298 K; Table S2: Photovoltaic parameters of reported BiI_3_ solar cells; Table S3. Photovoltaic parameters of reported Bi-based solar cells; Table S4: Photovoltaic parameters of 30 photovoltaic devices with CuSCN HTL and BiI_3_ or DMSO-coordinated BiI_3_ photoactive layers.
