# Supplementary material for: Bismuth Complex Controlled Morphology Evolution and CuSCN-Induced Transport Improvement Enable Efficient BiI3 Solar Cells
Source: Nanomaterials (Basel). 2022 Sep 8;12(18):3121. doi: 10.3390/nano12183121 (PMC9506543; doi:10.3390/nano12183121)
Supplement: Supplementary file 1 [file nanomaterials-12-03121-s001.zip › nanomaterials-1887727-supplementary.pdf]

*nanomaterials*

*Supplementary Materials*

# Bismuth Complex Controlled Morphology Evolution and CuSCN-Induced Transport Improvement Enable Efficient $BiI_3$ Solar Cells

**Zhangwei He [1], Runnan Yu [2], Wanrong Song [1], Yongshuai Gong [1], Hui Li [1] and Zhan'ao Tan [1,]***

[1] Beijing Advanced Innovation Center for Soft Matter Science and Engineering, Beijing University of Chemical Technology, Beijing 100029, China
[2] College of Materials Science and Engineering, Beijing University of Chemical Technology, Beijing 100029, China
* Correspondence: tanzhanao@mail.buct.edu.cn (Z.T.)

**Citation:** He, Z.; Yu, R.; Song, W.; Gong, Y.; Li, H.; Tan, Z. Bismuth Complex Controlled Morphology Evolution and CuSCN-Induced Transport Improvement Enable Efficient BiI3 Solar Cells. *Nanomaterials* **2022**, *12*, 3121. https://doi.org/10.3390/nano12183121

Academic Editor: Firstname Lastname

Received: 12 August 2022
Accepted: 6 September 2022
Published: 8 September 2022

**Publisher's Note:** MDPI stays neutral with regard to jurisdictional claims in published maps and institutional affiliations.

**Copyright:** © 2022 by the author. Licensee MDPI, Basel, Switzerland. This article is an open access article distributed under the terms and conditions of the Creative Commons Attribution (CC BY) license (https://creativecommons.org/licenses/by/4.0/).

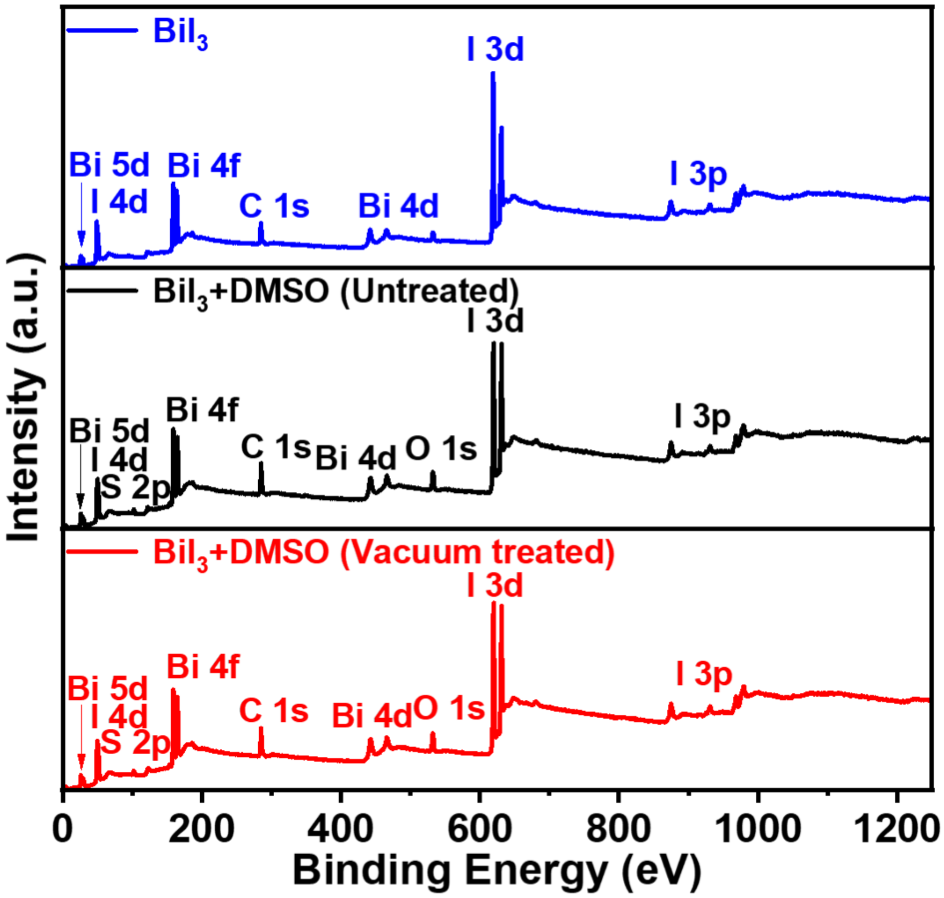


**Figure S1.** XPS spectra for $BiI_3$ film, and the DMSO-coordinated $BiI_3$ film without and with vacuum treatment.

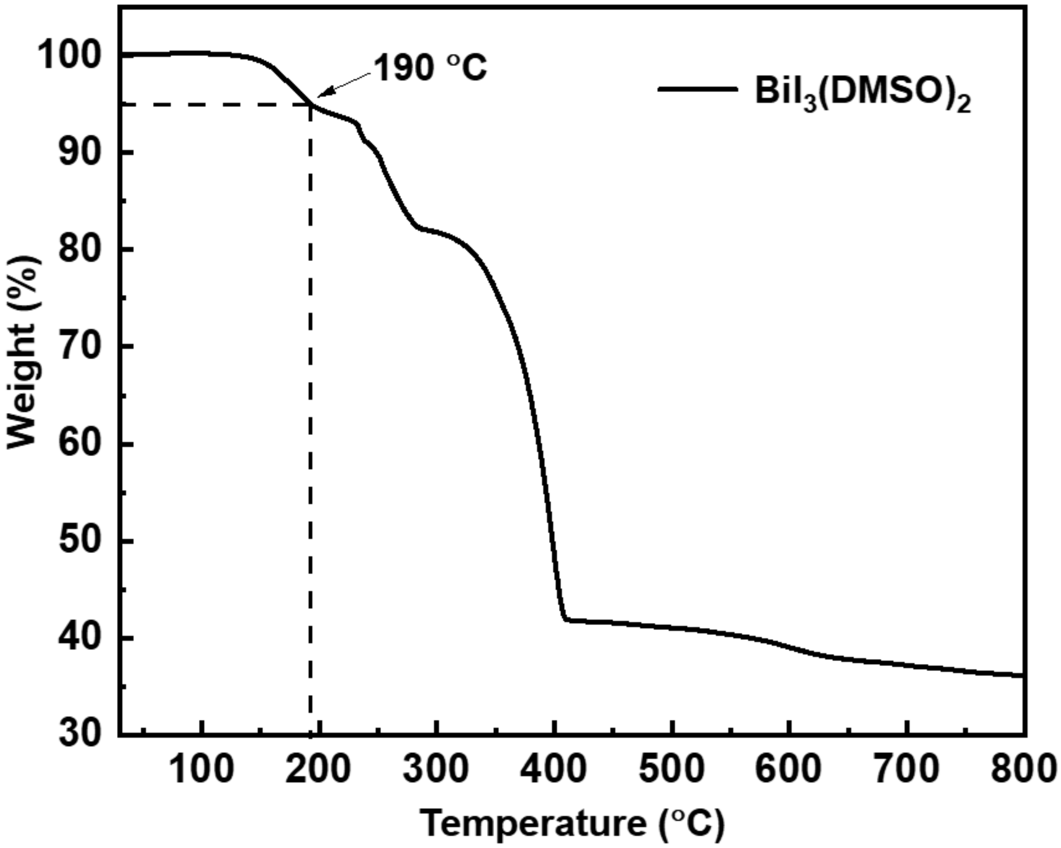


**Figure S2.** Thermogravimetric analysis of $BiI_3(DMSO)_2$ at (scan rate: 10 °C $min^{-1}$).

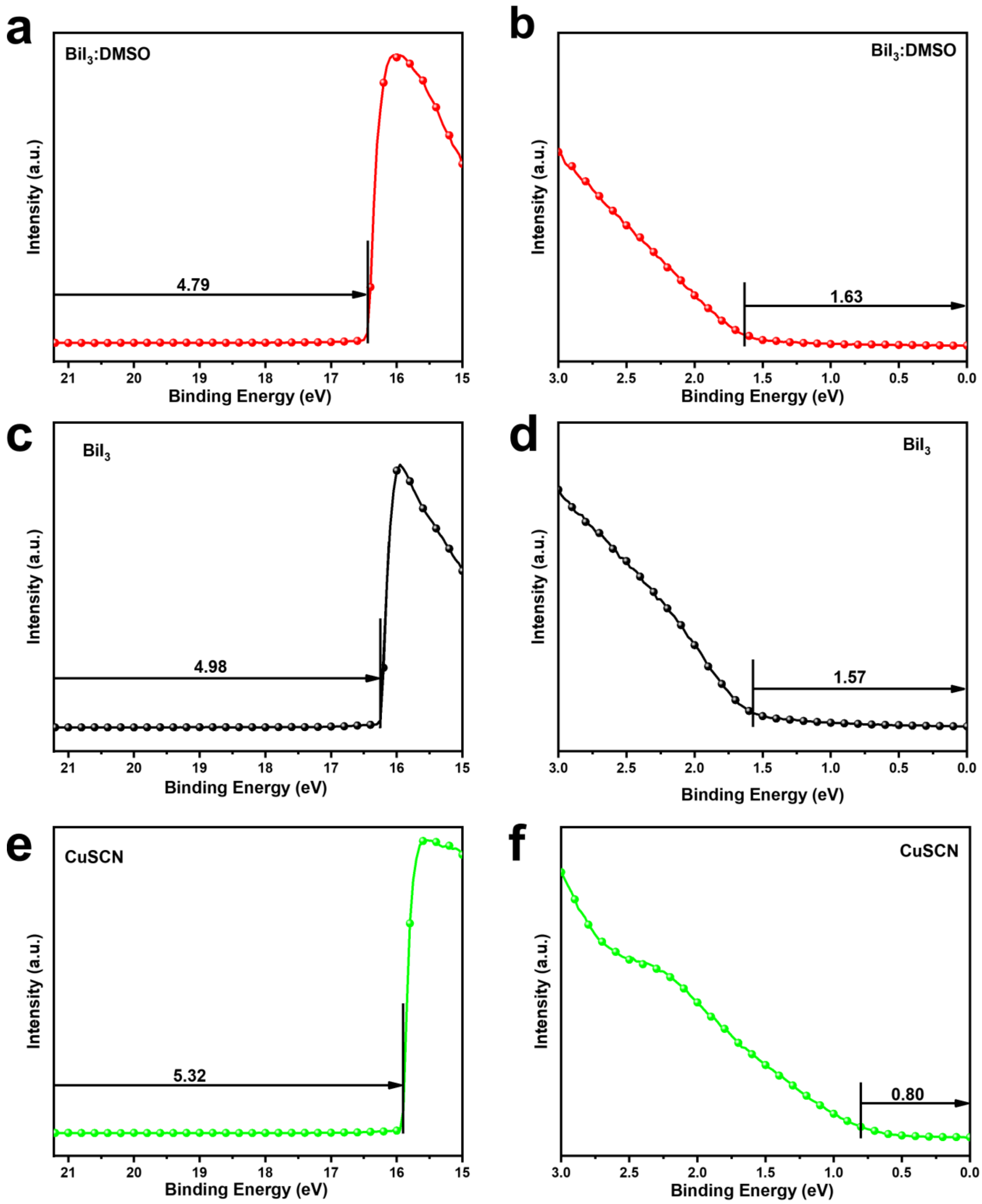


**Figure S3.** UPS spectra of (**a**,**c**,**e**) the high-binding energy secondary electron cut-off regions and (**b**,**d**,**f**) the valence band edge regions of $BiI_3$ film with and w/o DMSO-treated and CuSCN film, respectively.

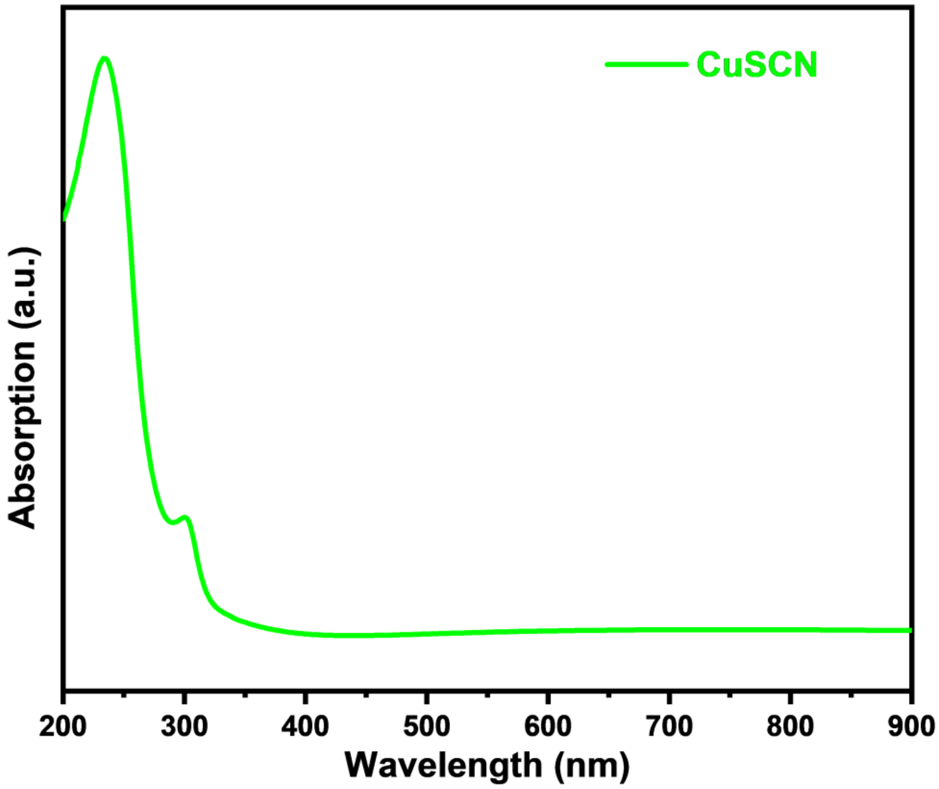


**Figure S4.** UV−vis absorption spectra of CuSCN.

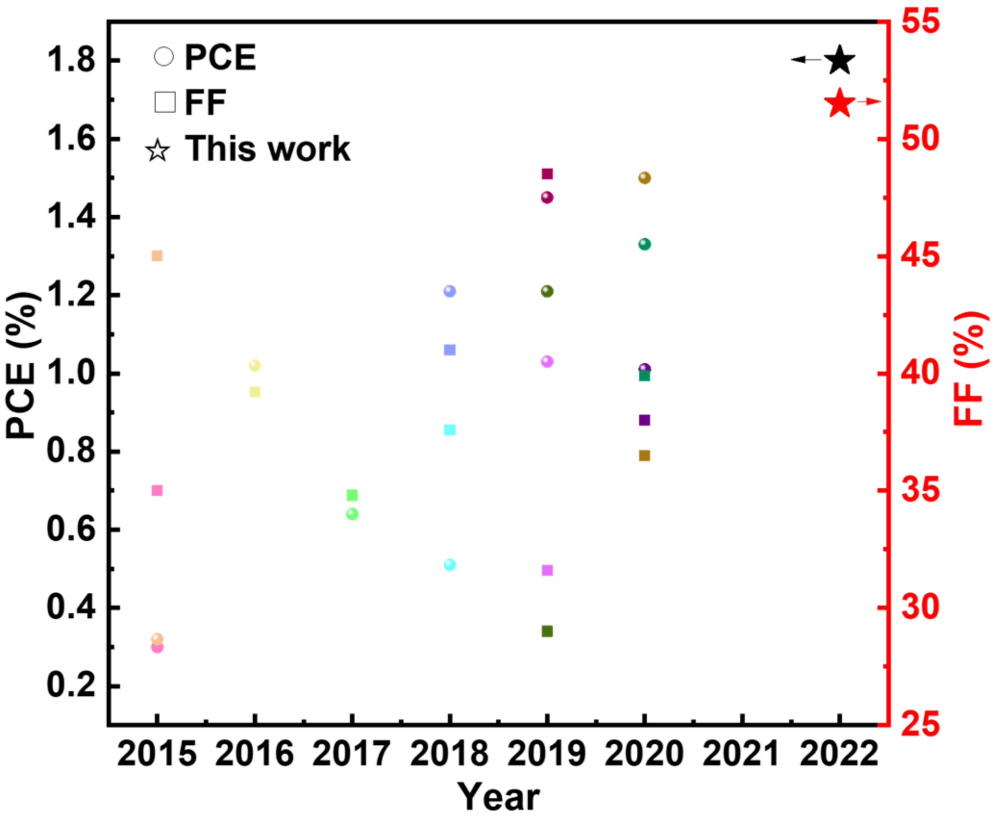


**Figure S5.** Comparison of PCE and FF values of this work with previous reported $BiI_3$ photovoltaic devices.

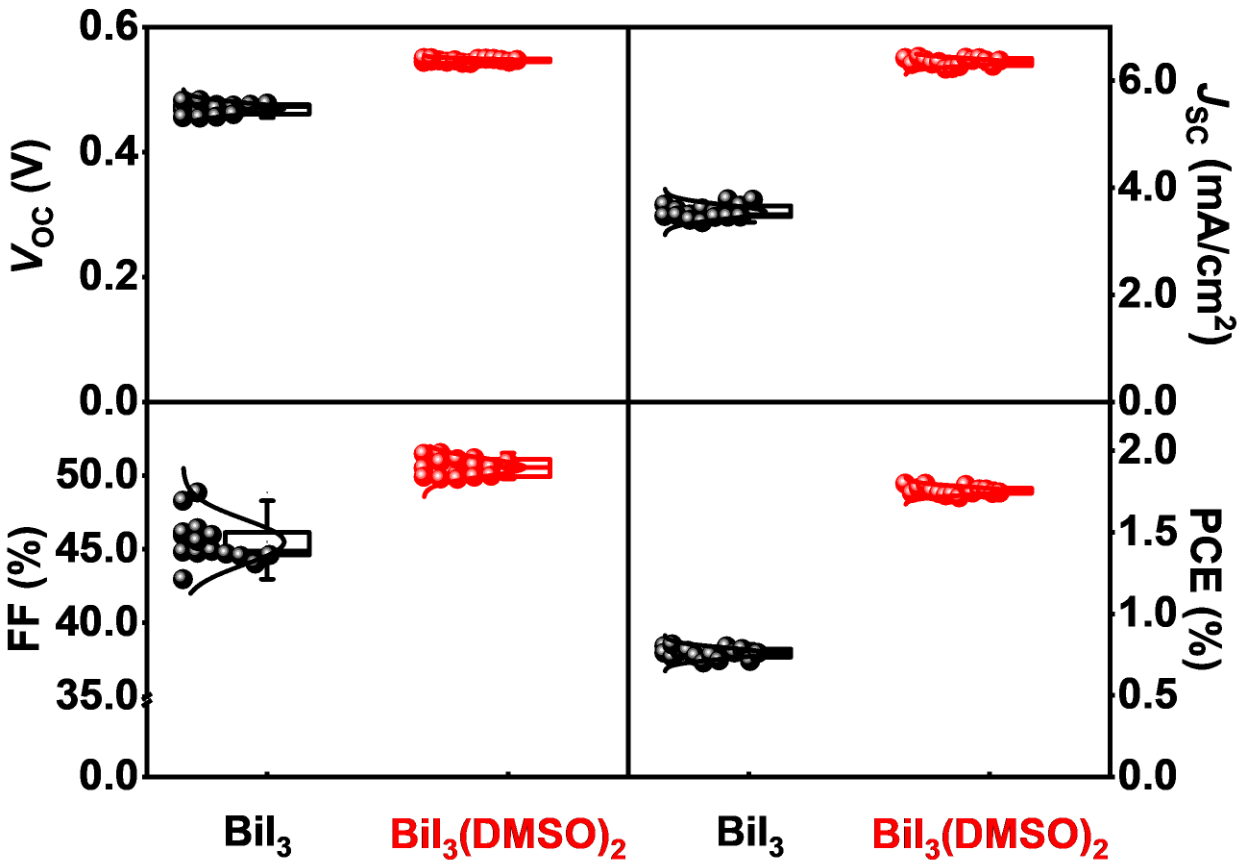


**Figure S6.** Statistical photovoltaic parameters obtained from 30 photovoltaic devices with CuSCN HTL and $BiI_3$ or DMSO-coordinated $BiI_3$ photoactive layers.

**Table S1.** Crystal data and structure refinement for $BiI_3(DMSO)_2$ at 298 K.

| | |
|---|---|
| Formula | $Bi_2I_6(DMSO)_4$ |
| Formula weight | 1491.87 |
| Temperature | 298 K |
| Wavelength | 0.71073 |
| Space group | P21/c |
| Crystal system | Monoclinic |
| Goodness-of-fit | 1.037 |
| Unit cell dimensions | a = 8.3303(6) Å, α = 92.275(5)°<br>b = 8.8638(6) Å, β = 101.467(6)°<br>c = 12.4967(8) Å, γ = 117.263(7)° |
| Volume | 794.75 (10) |
| Z | 1 |
| Density | 3.117 |
| F000 | 652.0 |
| Mu | 17.156 |
| R (reflections) = 0.0417 (3452) | $wR_2$ (reflections) = 0.1010 (4040) |

**Table S2.** Photovoltaic parameters of reported $BiI_3$ solar cells.

| Year | Device structure | $J_{SC}$ (mA/cm$^2$) | $V_{OC}$ (V) | FF (%) | PCE (%) | Reference |
|---|---|---|---|---|---|---|
| 2015 | FTO/$TiO_2$/$BiI_3$/PTAA/Au | 3.85 | 0.22 | 35.0 | 0.30 | [1] |
| 2015 | FTO/$TiO_2$/$BiI_3$/PIDT-DFBT/Au | 1.70 | 0.42 | 45.0 | 0.32 | [1] |
| 2016 | FTO/$TiO_2$/$BiI_3$/$V_2O_5$/Au | 7.0 | 0.36 | 39.2 | 1.02 | [2] |
| 2017 | ITO/PE-DOT/$NbSe_X$/$BiI_3$/$PC_{71}BM$/Ca/Al | 3.83 | 0.49 | 34.8 | 0.64 | [3] |
| 2018 | FTO/$TiO_2$/$BiI_3$/F8/Au | 5.28 | 0.61 | 37.6 | 1.21 | [4] |
| 2018 | FTO/$TiO_2$/$BiI_3$/Spiro-OMeTAD /Au | 3.69 | 0.33 | 41.0 | 0.51 | [5] |
| 2019 | ITO/$SnO_2$/BiSI/$BiI_3$/Spiro-OMeTAD /Au | 12.6 | 0.33 | 29.0 | 1.21 | [6] |
| 2019 | FTO/$SnO_2$/$BiI_3$/Spiro-OMeTAD /Au | 6.36 | 0.47 | 48.5 | 1.45 | [7] |
| 2019 | FTO/$TiO_2$/$BiI_3$/PTB7-Th/Au | 7.8 | 0.38 | 35.2 | 1.03 | [8] |
| 2019 | FTO/$TiO_2$/$BiI_3$/$V_2O_5$/Au | 3.43 | 0.26 | 33.3 | 0.30 | [9] |
| 2020 | ITO/PTAA/$BiI_3$/$PC_{61}BM$/BCP/Ag | 4.54 | 0.59 | 38.0 | 1.01 | [10] |
| 2020 | ITO/$V_2O_5$/$BiI_3$/ZnO/Ag | 5.57 | 0.60 | 39.9 | 1.33 | [11] |
| 2020 | ITO/PEDOT/$BiI_3$/$PC_{61}BM$/BCP/Ag | 8.76 | 0.47 | 36.5 | 1.50 | [12] |
| 2022 | ITO/CuSCN/$BiI_3$/$PC_{71}BM$/Ca/Al | 6.38 | 0.55 | 51.5 | 1.80 | This work |

**Table S3.** Photovoltaic parameters of reported Bi-based solar cells.

| Year | Device structure | $J_{SC}$ (mA/cm$^2$) | $V_{OC}$ (V) | FF (%) | PCE (%) | Reference |
|---|---|---|---|---|---|---|
| 2016 | FTO/c-$TiO_2$/mes-$TiO_2$/$AgBi_2I_7$/ P3HT/Au | 3.30 | 0.56 | 67.4 | 1.22 | [13] |
| 2017 | FTO/c-$TiO_2$/mes-$TiO_2$/ $MA_3Bi_2I_9$/Spiro-OMeTAD/Au | 0.94 | 0.51 | 61.0 | 0.31 | [14] |
| 2018 | FTO/c-$TiO_2$/$AgBi_2I_7$/spiroO-MeTAD/Au | 2.76 | 0.69 | 43.6 | 0.83 | [15] |
| 2018 | FTO/c-$TiO_2$/mes-$TiO_2$/$ZrO_2$/$CsBi_3I_{10}$/C | 4.75 | 0.46 | 69.1 | 1.51 | [16] |
| 2018 | FTO/c-$TiO_2$/mes-$TiO_2$/Ag-$BiI_4$/PTAA/Ag | 7.63 | 0.53 | 52.0 | 2.10 | [17] |
| 2019 | ITO/$NiO_x$/$Cs_3Bi_2I_9$/$PC_{61}BM$/Au | 3.42 | 0.74 | 51.0 | 1.26 | [18] |
| 2022 | ITO/CuSCN/$BiI_3$/$PC_{71}BM$/Ca/Al | 6.38 | 0.55 | 51.5 | 1.80 | This work |

**Table S4.** Photovoltaic parameters of 30 photovoltaic devices with CuSCN HTL and $BiI_3$ or DMSO-coordinated $BiI_3$ photoactive layers.

| Sample | $J_{SC}$ (mA/cm$^2$) | $V_{OC}$ (V) | FF (%) | PCE (%) |
|---|---|---|---|---|
| $BiI_3$ | 3.54 ± 0.13 | 0.47 ± 0.01 | 45.5 ± 1.5 | 0.75 ± 0.03 |
| $BiI_3$:DMSO | 6.35 ± 0.07 | 0.55 ± 0.01 | 50.6 ± 0.6 | 1.75 ± 0.02 |

## References


1. Lehner, A.J.; Wang, H.; Fabini, D.H.; Liman, C.D.; Hébert, C.-A.; Perry, E.E.; Wang, M.; Bazan, G.C.; Chabinyc, M.L.; Seshadri, R. Electronic structure and photovoltaic application of $BiI_3$. *Appl. Phys. Lett.* **2015**, *107*, 131109.
2. Hamdeh, U.H.; Nelson, R.D.; Ryan, B.J.; Bhattacharjee, U.; Petrich, J.W.; Panthani, M.G. Solution-Processed $BiI_3$ Thin Films for Photovoltaic Applications: Improved Carrier Collection via Solvent Annealing. *Chem. Mater.* **2016**, *28*, 6567–6574.

3. Lin, L.; Boopathi, K.M.; Ding, J.; Chu, C.W.; Chang, C.C. NbSex interlayers decrease interfacial recombination in $BiI_3$-Based hybrid solar cells. *FlatChem* **2017**, *5*, 18–24.
4. Tiwari, D.; Alibhai, D.; Fermin, D.J. Above 600 mV Open-Circuit Voltage $BiI_3$ Solar Cells. *ACS Energy Lett.* **2018**, *3*, 1882–1886.
5. Kulkarni, A.; Singh, T.; Jena, A.K.; Pinpithak, P.; Ikegami, M.; Miyasaka, T. Vapor Annealing Controlled Crystal Growth and Photovoltaic Performance of Bismuth Triiodide Embedded in Mesostructured Configurations. *ACS Appl. Mater. Inter.* **2018**, *10*, 9547–9554.
6. Yoo, B.; Ding, D.; Marin-Beloqui, J.M.; Lanzetta, L.; Bu, X.; Rath, T.; Haque, S.A. Improved Charge Separation and Photovoltaic Performance of $BiI_3$ Absorber Layers by Use of an In Situ Formed BiSI Interlayer. *ACS Appl. Energy Mater.* **2019**, *2*, 7056–7061.
7. Zhu, Y.; Zhang, Q.; Kam, M.; Poddar, S.; Gu, L.; Liang, S.; Qi, P.; Miao, F.; Fan, Z. Vapor phase fabrication of three-dimensional arrayed $BiI_3$ nanosheets for cost-effective solar cells. *InfoMat* **2019**, *2*, 975–983.
8. Ma, S.; Yang, Y.; Liu, C.; Cai, M.; Ding, Y.; Tan, Z.A.; Shi, P.; Dai, S.; Alsaedi, A.; Hayat, T. Vertically Oriented $BiI_3$ Template Featured $BiI_3$/Polymer Heterojunction for High Photocurrent and Long-Term Stable Solar Cells. *ACS Appl. Mater. Inter.* **2019**, *11*, 32509–32516.
9. Hamdeh, U.H.; Nelson, R.D.; Ryan, B.J.; Panthani, M.G. Effects of Solvent Coordination Strength on the Morphology of Solution-Processed $BiI_3$ Thin Films. *J. Phys. Chem. C* **2019**, *123*, 13394–13400.
10. Pandian, M.G.M.; Khadka, D.B.; Shirai, Y.; Umedov, S.; Yanagida, M.; Subashchandran, S.; Grigorieva, A.; Miyano, K. Effect of solvent vapour annealing on bismuth triiodide film for photovoltaic applications and its optoelectronic properties. *J. Mater. Chem. C* **2020**, *8*, 12173–12180.
11. Wang, Y.; Shi, X.; Wang, G.; Tong, J.; Pan, D. All-Inorganic and lead-Free $BiI_3$ thin film solar cells by iodization of BiSI thin films. *J. Mater. Chem. C* **2020**, *8*, 14066–14074.
12. Kang, J.; Chen, S.; Zhao, X.; Yin, H.; Zhang, W.; Al-Mamun, M.; Liu, P.; Wang, Y.; Zhao, H. An inverted $BiI_3$/PCBM binary quasi-Bulk heterojunction solar cell with a power conversion efficiency of 1.50%. *Nano Energy* **2020**, *73*, 104799.
13. Kim, Y.; Yang, Z.; Jain, A.; Voznyy, O.; Kim, G.-H.; Liu, M.; Quan, L. N.; García de Arquer, F. P.; Comin, R.; Fan, J. Z.; et al. Pure Cubic-Phase Hybrid Iodobismuthates $AgBi_2I_7$ for Thin-Film Photovoltaics. *Angew. Chem. Int. Ed.* **2016**, *55*, 9586–9590
14. Kulkarni, A.; Singh, T.; Ikegami, M.; Miyasaka, T. Photovoltaic enhancement of bismuth halide hybrid perovskite by N-methyl pyrrolidone-assisted morphology conversion. *Rsc Adv.* **2017**, *7*, 9456-9460
15. Shao, Z.; Le Mercier, T.; Madec, M. B.; Pauporté, T. $AgBi_2I_7$ layers with controlled surface morphology for solar cells with improved charge collection. *Mater. Lett.* **2018**, *221*, 135-138
16. Shin, J.; Kim, M.; Jung, S.; Kim, C. S.; Park, J.; Song, A.; Chung, K.-B.; Jin, S.-H.; Lee, J. H.; Song, M. Enhanced efficiency in lead-free bismuth iodide with post treatment based on a hole-conductor-free perovskite solar cell. *Nano Res.* **2018**, *11*, 6283-6293
17. Lu, C.; Zhang, J.; Sun, H.; Hou, D.; Gan, X.; Shang, M.-h.; Li, Y.; Hu, Z.; Zhu, Y.; Han, L. Inorganic and Lead-Free $AgBiI_4$ Rudorffite for Stable Solar Cell Applications. *ACS Appl. Energy Mater.* **2018**, *1*, 4485-4492
18. Khadka, D. B.; Shirai, Y.; Yanagida, M.; Miyano, K. Tailoring the film morphology and interface band offset of caesium bismuth iodide-based Pb-free perovskite solar cells. *J. Mater. Chem. C* **2019**, *7*, 8335-8343
